# Sensing Native Protein Solution Structures Using a Solid-state Nanopore: Unraveling the States of VEGF

**DOI:** 10.1038/s41598-018-19332-y

**Published:** 2018-01-17

**Authors:** Nitinun Varongchayakul, Diana Huttner, Mark W. Grinstaff, Amit Meller

**Affiliations:** 1 Departments of Biomedical Engineering, Boston University, Boston, 02215 Massachusetts USA; 2 Department of Chemistry, Boston University, Boston, 02215 Massachusetts USA; 3 School of Medicine, Boston University, Boston, 02215 Massachusetts USA; 4 Faculty of Biomedical Engineering, Technion – Israel Institute of Technology, Haifa, 32000 Israel

## Abstract

Monitoring individual proteins in solution while simultaneously obtaining tertiary and quaternary structural information is challenging. In this study, translocation of the vascular endothelial growth factor (VEGF) protein through a solid-state nanopore (ssNP) produces distinct ion-current blockade amplitude levels and durations likely corresponding to monomer, dimer, and higher oligomeric states. Upon changing from a non-reducing to a reducing condition, ion-current blockage events from the monomeric state dominate, consistent with the expected reduction of the two inter-chain VEGF disulfide bonds. Cleavage by plasmin and application of either a positive or a negative NP bias results in nanopore signals corresponding either to the VEGF receptor recognition domain or to the heparin binding domain, accordingly. Interestingly, multi-level analysis of VEGF events reveals how individual domains affect their translocation pattern. Our study shows that careful characterization of ssNP results elucidates real-time structural information about the protein, thereby complementing classical techniques for structural analysis of proteins in solution with the added advantage of quantitative single-molecule resolution of native proteins.

## Introduction

Cytokines are small signaling proteins that mediate cell-cell communication involved in infection, immune response, inflammation, trauma, transplantation, degenerative age processes, and cancer^1–4^. Many cytokines have emerged as important therapeutic targets or as predictive or prognostic biomarkers. Nowadays, the gold standard method used to quantify cytokines in solution is enzyme-linked immunosorbent assay (ELISA), which relies on target recognition based on antibody specificity and sensitivity^5^. Vascular endothelial growth factor (VEGF), is one of the most well studied cytokines due to its pivotal role in stimulating vascularization and angiogenesis, in normal as well as in patho–physiological processes, such as cancer^6–12^. VEGF comprises of a family of proteins: VEGFA, VEGFB, VEGFC, VEGFD, and placental growth factor. The principle mediators of tumor angiogenesis are the soluble VEFG_121_ and VEGF_165_, which are both isoforms of VEGFA, produced by alternative splicing. VEGFA isoforms are important therapeutic targets for inhibition of angiogenesis and cancer treatment, although their role as predictive biomarkers for certain cancer types has yet to be established^13^. The longer isoform, VEGF_165_, is the predominant form. Since VEGF_165_ is the focus of our study, for simplicity it will be referred to as VEGF from now onwards. VEGF consists of two domains: the VEGF_1–110_ receptor recognition domain^14^ and the VEGF_111–165_ heparin binding domain^15^, linked by a plasmin cleavable flexible segment^16,17^. The disulfide bonds of VEGF stabilize the secondary and tertiary structures as well as control the self-assembly of VEGF to form homodimers^18^. Thus, obtaining tertiary and quaternary structural information on VEGF in solution (both statically and dynamically) is of medical relevance and interest. Herein, we investigate the conformational landscape of VEGF multimers using solid-state nanopores (ssNPs). Specifically, by exploring the effect of reducing agents and reaction with plasmin enzyme, we show that VEGF dimerization dynamics and structure are directly probed by the nanopore, obviating the need for antibodies or specific protein labeling. Furthermore, we relate the step-like features present in the ion-current traces during VEGF translocation to the structural domains of VEGF.

Nanopore (NP) sensing is an advanced technique for biomolecular characterization at the single-molecule level that uses either a biological or a solid-state NP^19,20^. In a NP setup, an applied electrical voltage produces a strongly converging electric field near the pore’s vicinity. This voltage produces an ion current through the pore that can be measured using a sensitive electrometer. Charged biomolecules are subject to an electrophoretic force that draws them towards the NP^21^. Upon capture, the biomolecule electrophoretically threads through the pore partially blocking the ion current. NPs have been used to characterize proteins by resolving their size^22–25^, charge^26^, post-translational modifications^27–30^, specific interactions with an antibody or DNA^31–33^, aggregated state^34^, or a combination of them^35,36^. However, NP detection of multimeric proteins has remained elusive to date, partly due to the multiple conformations that these complexes can exhibit in solution, which leads to complex ion current signatures and broad translocation dwell-time distributions characterizing this process.

## Results and Discussion

### VEGF monomer/dimer population identification

We chose VEGF as a model protein due to its clinical importance on the one hand, and due to its interesting and well-characterized structure, on the other hand. Purified recombinant VEGF_165_ was analyzed using locally thinned silicon-nitride (SiN_x_) membranes with a thickness of 12 nm to improve the spatial resolution of the detection^25,32,37^. Furthermore, the pore used were ~5.5 nm in diameter, slightly larger than the VEGF’s diameter (4.12 nm and 5.20 nm for VEGF monomer and dimer, respectively, estimated by their hydrodynamic radii, as shown in the Supporting Information file). The pore’s geometry was confirmed by conductance measurement as described in the SI. In the pore size range *d*_*protein*_*/d*_*pore*_ >0.8 further enhancing the interactions between the mobile molecule and the pore’s wall^25,38^. Notably, we have chosen the applied voltage and pH values in order to shift the mean dwell time of the ion current blockage events much above the minimum resolution of our system (~10 µs) hence permitting the detection of VEGF translocations. To confirm that the observed events represent protein translocations and not collisions, we measured the characteristic dwell-time of VEGF ion current blockade events at pH 7.2 as a function of the applied voltage. Our results (Figure SI5) show that as the voltage amplitude increases from 300 mV to 700 mV, the mean residence time (obtained from tail-fitting the dwell time histogram with the exponential functions) decreases from 391 ± 55 µs to 56 ± 4 µs. Notably, if the observed events were due to protein collisions, increasing voltage should result in *longer* dwell time^39,40^.

**Figure 1:**
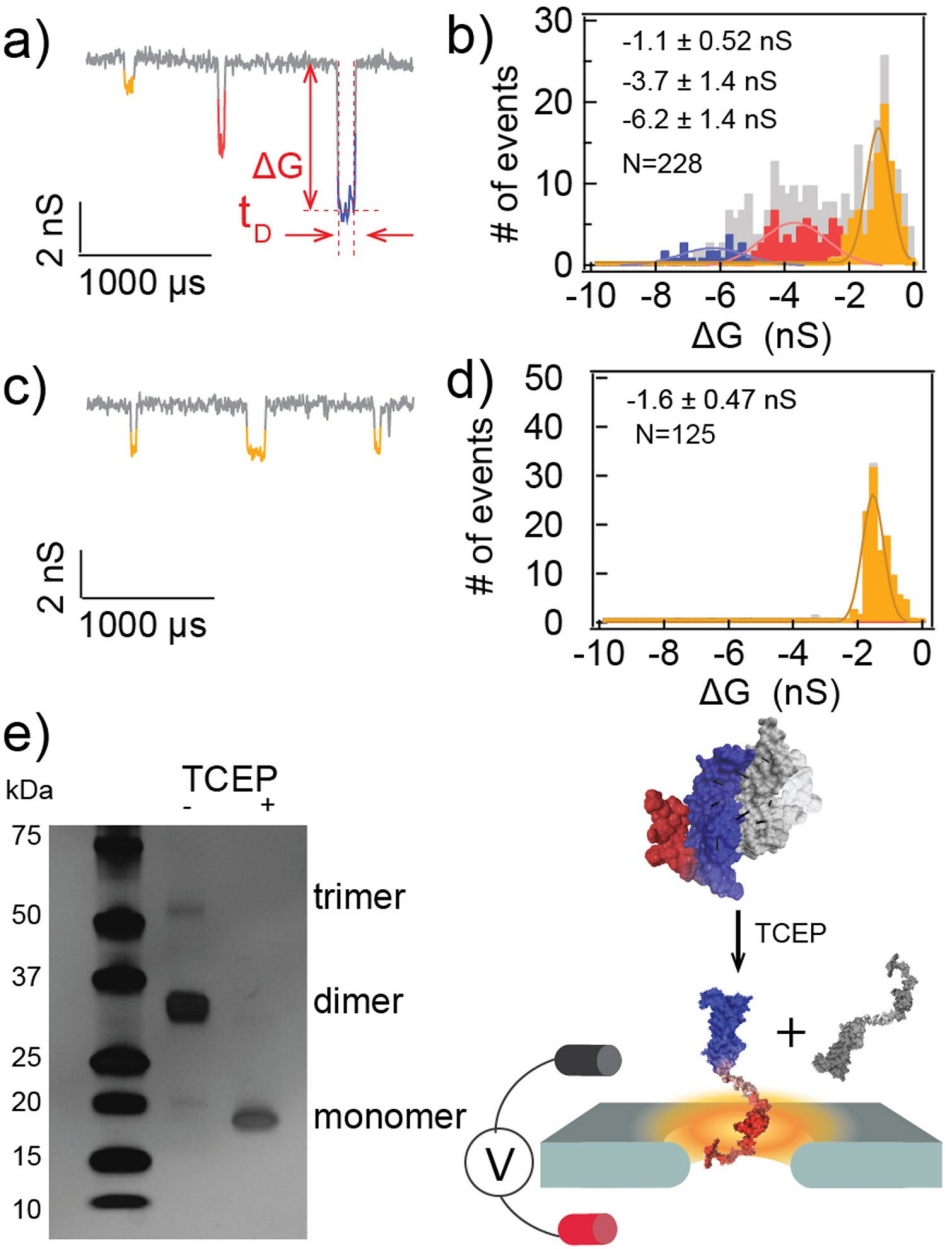
Reduction of VEGF results in altered oligomeric states, reflected by different ssNP translocation populations’ distribution. (**a**,**b**) representative translocation events of 20 nM VEGF and the amplitude histogram (V = +500 mV). Orange, red, and blue represent groups A, B and C, respectively. The grey bar represents segmented levels from multi-level events. The scatter plot of the event histogram *vs*. event amplitude is shown in the SI. (**c**,**d**) 15 minutes after adding 0.5 mM of TCEP directly into the NP chamber with 20 nM VEGF. This experiment was done in the same NP as of (**a**,**b**). (**e**) SDS gel electrophoretic image of VEGF before and after reduction (a full-length gel shown in Figure SI1). Cartoons illustrate native dimeric VEGF (top) and monomeric VEGF (bottom) as a result of TCEP reduction.

Typical events from a 20 nM VEGF solution at pH 7.6 are displayed in Fig. 1a (V = +500 mV). Additional events are showed in Figure SI3. This solution pH was chosen because VEGF isoelectric point is ≈7.45^41^, and thus, under a positive applied bias the protein would likely translocate from the *cis* to the trans chamber. Approximately 85% of the events (N_total_ = 228) exhibited a single current event amplitude level (defined by the change in the pore conductance before and during the translocation event), which could be further classified into three groups or populations according to their average event amplitudes (Fig. 1b, displayed in different colors). When fit by Gaussian functions, the three groups possessed a mean ± standard deviation of −1.1 ± 0.52 nS (Group A, marked in orange, N_A_ = 86); −3.7 ± 1.4 nS (Group B, marked in red, N_B_ = 79) and −6.2 ± 1.4 nS (Group C, marked in blue, N_C_ = 30) (Fig. 1b). Additionally, about 15% of the events contained multiple blockage levels within one event.

Immediately following this experiment, and using the same NP, the VEGF solution was treated with 0.5 mM tris(2-carboxyethyl)phosphine (TCEP) to chemically reduce the protein’s disulfide bonds. As shown in Fig. 1c-d, the subsequent NP measurements revealed a sharp shift in the entire population. The three distinctive event amplitude groups observed in the absence of the reducing agent (Fig. 1a-b) converged into a single group (92%) at −1.6 ± 0.47 nS (N_total_ = 125, Fig. 1c-d) labeled in orange color. This group had similar amplitude and dwell time as the events of group A, prior to TCEP treatment.

The convergence of the three event populations into a single, lower-amplitude, population after TCEP treatment led us to speculate that these three distinctive groups, observed at pH 7.6, represent the VEGF monomer as well as the dimers and trimers connected via disulfide bonds. Upon TCEP reduction, the majority of the higher order structures are reduced to give the monomeric form, which remains intact due to additional intra-hydrophobic interactions^16,42^. In support of this conclusion, a VEGF solution was treated with TCEP and analyzed by sodium dodecyl sulfate polyacrylamide (SDS-PAGE) gel electrophoresis. The bands corresponding to the dimers and trimers of VEGF were no longer present and only the single band of the VEGF monomer was observed (Fig. 1e).

### VEGF population dependence on pH and concentration

**Figure 2:**
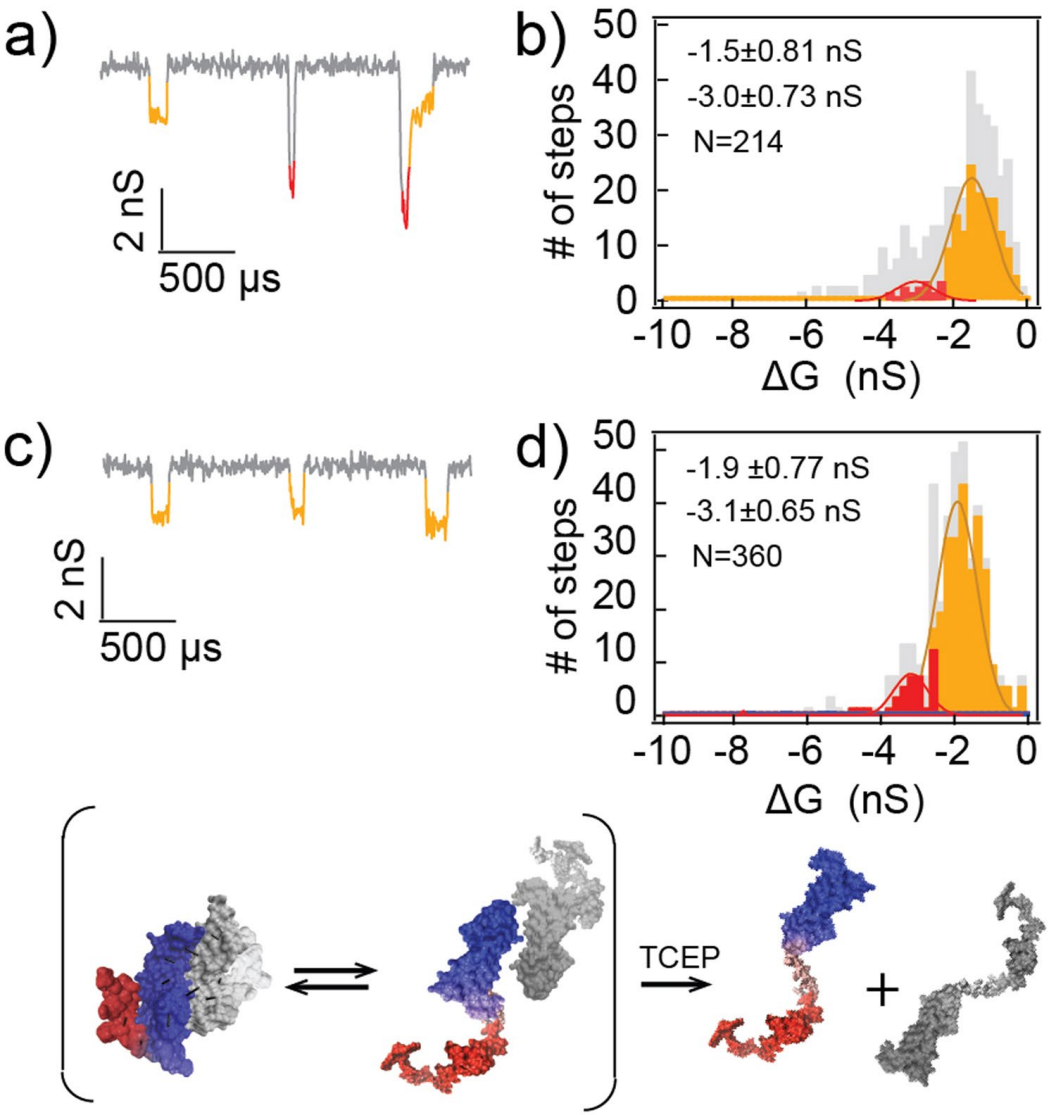
VEGF translocation at pH 7.2. (**a**,**b**) representative translocation events of 20 nM VEGF pH 7.2 (V = −500 mV) and its amplitude histogram. Orange and red represent group A and B, respectively. The grey color represents segmented levels from multi-level events. (**c**,**d**) 20 minutes after adding 0.5 mM of TCEP. Cartoons illustrate the interpretation that the monomers exist in the solution, and are reduced after adding TCEP.

When the pH of the VEGF solution was lowered to 7.2, below its pI, VEGF signals significantly changed. First, as expected, applying a positive bias no longer afforded protein translocation as the protein is positively charged, while applying a negative bias (i.e., V = −500 mV) resulted in series of ion current blockades likely due to protein translocations (Fig. 2a). Second, the VEGF events (N_total_ = 214) were characterized by only two amplitude populations, A (55%) and B (12%) (Fig. 2b). At the same time, we observed an increase in the fraction of translocation events that exhibited multiple blockage levels within one event (33%, as opposed to 15% in the pH 7.6 case, shown as a grey histogram in Fig. 2b). Further analysis of these multi-level events revealed that many of the multiple blockage levels showed a discernable pattern of B and A transition (28%, Fig. 2a third event, additional events are provided in the SI Figure SI4). As before, reduction of the solution using TCEP shifted the population towards single level A events (−1.9 ± 0.70 nS, 71%, N_total_ = 360), consistent with monomeric VEGF translocations (Fig. 2c-d).

Multi-level protein translocation have been previously observed and rationalized as protein trapping or rotating inside the NP^35,43^, resulting in a fluctuation of the current. In our experimental design, we selected the pore’s geometry to be smaller than the predicted largest dimension of monomeric VEGF (see figure SI8 for VEGF’s crystal structure) to restrict rotational movement. Furthermore, among the multi-level events, 81% of them showed explicitly bi-level transition from B to A (57 out of 70 events). This predominantly asymmetrical pattern indicates that the protein may not freely rotate in the NP (leading to a random pattern). Other researchers have proposed that the multi-level translocations originate from a complete unfolding and linearization of the protein^24,44–48^. Our theoretical calculations showed that the expected blocked level of a completely linearized peptide will be undetectable as its signal will be below the noise threshold (~0.2 nS, see SI) and not similar to the current in level A. Hence, a plausible explanation is that, rather than complete unfolding, the B to A transition originates from protein shearing or partial dissociation of the VEGF dimer to its monomeric form. We cannot rule out either scenario, but it is more likely to be predominantly protein shearing since, in most events, level B is immediately followed by level A without a current jump to the open pore level. In contrast, if partial dissociation events were predominant, we should have observed B to A events, where a small excursion of the current to the open pore level is followed by an A event, and this is not the case. Furthermore, we observed that this multi-step behavior is dependent on the pH (i.e. more fraction of B/A events observed at pH 7.2 as oppose to at pH 7.6). This is in agreement with the finding that disulfide bond stability is also pH dependent^49^.

**Figure 3:**
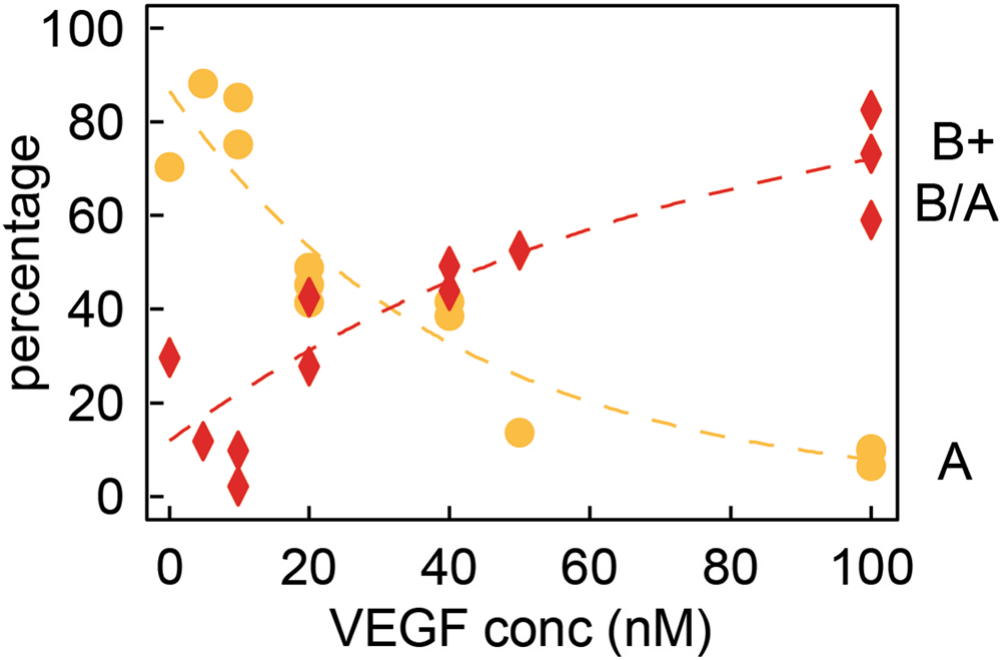
NP sensing detects changes in the steady state equilibrium of VEGF oligomeric states. Percentage of group A translocation events as well as group B and B/A translocation events. The experiments were done in ~5–6.5 nm pore at pH 7.2 (V = −500 mV). The dashed lines are guide to the eye. See Table SI1 for the complete experimental conditions and statistics.

To further investigate the dependencies of VEGF dimer and higher-order oligomer signals, the concentration of VEGF was varied from 0.2 nM to 100 nM, which covers the physiological relevant concentration range of VEGF^10^. As the concentration of the protein increases, the ratio of monomer structures shifted towards the dimeric state (i.e., a greater percentage of B and multi-level B/A translocations as opposed to A only translocations). As shown in Fig. 3, the percentage of population A decreased from 94% at 0.2 nM to 10% at 100 nM, while the percentages of level B and B/A increased from 6% to 59%.

### VEGF’s domain characterization

**Figure 4:**
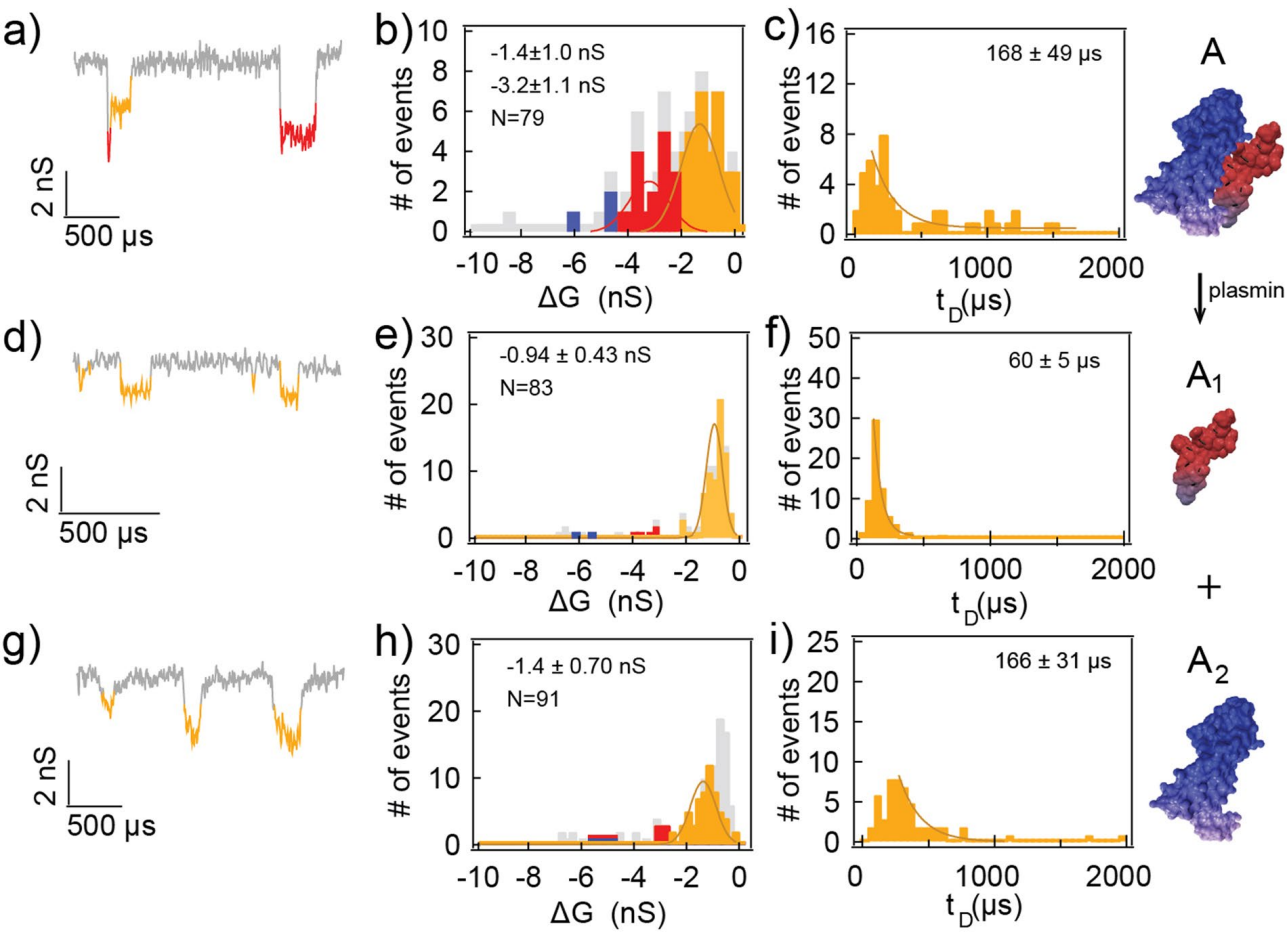
Plasmin-catalyst VEGF cleavage experiment. Cartoons represent the VEGF in native state (top panel), and upon reduction with plasmin, which results in positively charged VEGF_111–165_ domain (middle panel) and negatively charged VEGF_1–110_ domain (bottom panel). (**a**,**b**) Representative events and the amplitude histogram of 20 nM VEGF at pH 7.2. Orange and red represent group A and B, respectively. (**c**) The dwell time histogram of group A events, tail fit by an exponential function to obtain the characteristic dwell time. (**d**,**e**) Translocation events after 15 min of adding plasmin. (**f**) The dwell time histogram of group A_1_ events. (**g**,**h**) Translocations were also observed under a positive bias. (**i**) The dwell time histogram of group A_2_ events.

As mentioned in the introduction, the VEGF receptor recognition (VEGF_1–110_) and the heparin binding (VEGF_111–165_) domains are connected by a linker sensitive to cleavage by plasmin. Plasmin plays a critical role in the VEGF signaling pathway by releasing the VEGF receptor recognition domain from the heparin binding domain in extracellular matrix, thereby promoting its binding to the VEGF receptor^17^. To study this biochemical reaction at the single molecule level, plasmin (10 nM) was added to a solution of VEGF (20 nM) at pH 7.2, and the translocation events were monitored (Fig. 4). The events profile shifted from three groups (Fig. 4a–c) to a single-level amplitude population at −0.94 ± 0.43 nS (87%, N_total_ = 83, Fig. 4d–f), which we denoted group A_1_. These events likely corresponded to the translocation of the positively charged VEGF_111–165_ domain. When the applied bias was switched to a positive bias, single-level events of the negatively charged VEGF_1–110_ domain were predominantly measured with an amplitude of −1.4 ± 0.70 nS (85%, N_total_ = 91, Fig. 4g–i), marked as group A_2_. Importantly, there were no recorded events that correspond to the plasmin translocation through the pore. This result is expected since the 83-kDa plasmin (~9 nm, from pdb file ID 4DUU, see Figure SI8-SI9) is significantly larger than our 4.5 nm pore.

**Figure 5:**
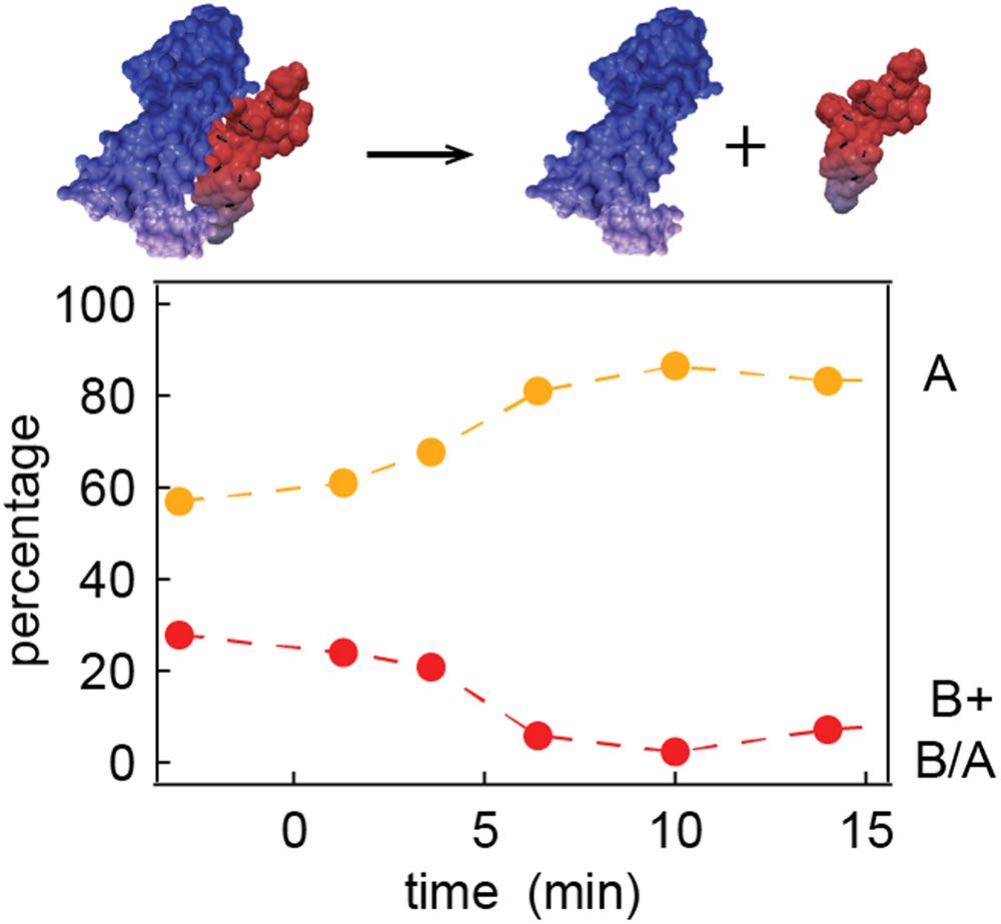
Kinetics of plasmin-catalyzed VEGF dissociation probed by NP. At t = 0 min, 10 nM of plasmin was added into the NP chamber containing 20 nM VEGF and then the translocation behaviour was tracked as a function of time (V = −500 mV). A ~4.5 nm NP was used for the entire experiment.

Next, we characterized the enzymatic catalysis rate of VEGF by plasmin by measuring the events’ rate at different time points after addition of plasmin (Fig. 5). The reaction was probed under a negative bias. Prior to the addition of plasmin (t< 0), 57% of the events were contained in group A. Upon addition of plasmin, the percentage of the A and A_1_ events, corresponding to the positively charged positively charged VEGF_111–165_ domain, increased to 87% after 10 min (orange line) and remained unchanged. In contrast, the percentage of group B and B/A events decreased from 28% to less than 10% after 10 min (red line). When plasmin cleaved the VEGF dimer, the positively charged VEGF_111–165_ domain was detached from the negatively charged VEGF_1–110_ receptor recognition domain (A_2_). Hence, under the applied negative bias the A_2_ domain are not likely to translocate through the pore. This results in a reduction in the number of group B and B/A events. We further determined that the half-life of VEGF_1–165_, which corresponds to group B blockage, is in a timescale of 10 min under our experimental condition, (20 nM VEGF, 10 nM plasmin). The calculated rate constant of the reaction in these conditions is 1.6 · 10^5^ M^−1^s^−1^, which is a typical rate constant for a protease reaction (see SI for detailed calculation and summarized data).

Under a positive bias, the likely translocations from the plasmin experiment arose from passage of the negatively charged VEGF_1–110_ receptor recognition domain A_2_. Interestingly, the population amplitude resembled the translocation of the monomeric VEGF (group A, Fig. 4b). This result suggests that the blockage level of monomeric VEGF is dominated by the blockage level of its VEGF_1–110_ domain. This interpretation is also consistent with the results from the TCEP reduction experiments where the disulfide bonds between dimers were reduced, however, VEGF_1–110_’s secondary structure remained intact due to the hydrophobic interactions between the four chains^18^.

Although the presence of the VEGF_111–165_ domain did not contribute to the blockage level, it did alter the overall charge and the NP dwell time of the protein. The dwell times were 168 ± 49 µs, 50 ± 5 µs to 166 ± 31 µs, for VEGF_1–165_, VEGF_1–110_, and VEGF_111–165_, respectively (Fig. 4c,f,i). The full monomeric VEGF translocated slower than the cleaved VEGF_111–165_ domain because the overall charge was closer to zero. These results show that the NP can be used to differentiate between the alternative splicing isomeric state of VEGF; VEGF_165_ and VEGF_121_ simply by switching bias.

## Summary

Choosing VEGF as a model protein, we were able explore and elucidate the potential in NP sensing of proteins. Our analysis demonstrates that careful analysis of the NP ion-current signals can be harnessed to better characterize and quantify structural features of proteins in solution. By manipulating the assay conditions, changing the pH of the analyte, using either reducing or non-reducing conditions and alternating the electrical bias, we were able to monitor changes in the native oligomeric states of VEGF. The NP translocation results obtained at pH 7.6, above the pI of VEGF, were expected and matched the observed oligomeric states of VEGF on SDS-PAGE under reducing and non-reducing conditions. However, our results, which were obtained at pH 7.2, below the pI of VEGF, were surprising and emphasized how slight pH change can affect the structure of the protein in solution. By probing VEGF at a range of concentrations, we were able to reveal the relative amounts of monomer to higher oligomeric states in real time. Hence, NP sensing can be used to complement the structural information obtained by classical methods, such as NMR, analytical ultracentrifugation and solution scattering.

VEGF has a well-established role in promoting angiogenesis and it is an important therapeutic target for cancer treatment. However, the exact roles of the different VEGF isoforms, and particularly that of the predominant VEGF_121_ and VEGF_165_, are not entirely understood. This is mainly due to lack of isoform-specific antibodies used in ELISA, the gold standard quantification method available nowadays. By carefully analyzing the NP translocation patterns of VEGF domains cleaved by plasmin, we developed a single-molecule method to specifically identify and quantify each isoform. Experiments are currently ongoing to study other biologically significant cytokines using our approach. Specifically, to determine if different proteins exhibit unique translocation signature/profile as a function of pH, applied voltage and polarity. A combination of NP signature with antibody or aptamer might be useful in separating a target protein from solution mixture. In the future, this method may be utilized in the clinic to analyze patients’ derived samples, and to better assess cytokines’ potential as predicative biomarkers for anti-angiogenic therapy.

## Methods

### Nanopore fabrication and drilling

Nanopore chips were fabricated as previously described.^32^ In brief, silicon wafer coated with 500 nm SiO_2_ (Virginia Semiconductors) and low-stress LPCVD amorphous silicon nitride (SiN_x_: 50 nm, Cornell CNF) were used. The SiN_x_ was locally thinned to 7–12 nm in ~2 µm circular wells, using a lithographic-mask whole wafer protection followed by reactive-ion etching. Then the silicon wafer was etched through with KOH, leaving a free-standing membrane of SiN_x_ with a thinned area in the center of the window. NPs were drilled through SiN_x_ using highly focused transmission electron microscope (JEOL 2010 FEG) to sputter away materials from the thinned membrane. Pores of 4–6 nm could be formed within 1 minute of focusing the beam. Unless otherwise stated, we used 5.5 nm diameter ssNP fabricated in a 12-nm thick SiN_x_ membrane. Since small pore-to-pore variations in diameters can bias the classification of translocation events amplitude, we performed each set of experiments using the same NP device and discarded data in which pores expanded during a given measurement.

### Protein sample

VEGF used in this study was a recombinant human protein purchased from Biological Industries. The purity of the sample was >98%. The stock powder protein was reconstituted with MilliQ water (Millipore), 10% glycerol and 1 mM of dithiothreitol (DTT) to the stock concentration of 20 µM and then stored at −80 °C until use. Upon usage, this stock solution was then mixed with the nanopore buffer to obtain the final concentration without any further additives. The protein concentration inside the nanopore chamber ranged from 0.5–100 nM.

### DNA sample

The linear 5406 bps DNA fragments were amplified from pET28b by PCR. The DNA was separated on 0.7% agarose TAE gel, excised and extracted from the gel using a PCR cleanup kit (Promega). The DNA was further purified via ethanol precipitation, aliquoted at 0.1 pmol per tube and dried using the speedvac. It was stored at −20 °C. Prior to use, it was rehydrated in 10 microliter milliQ water and incubated at 37 °C for 1 hour at 300 rpm.

### Nanopore electrical sensing

Prior to the experiment, each NP was treated with boiled piranha solution (70%H_2_SO_4_: 30%H_2_O_2_) to remove organic residues and make the NP interior walls hydrophilic, followed by intensive washing in MilliQ water. Upon the experiment, the nanochips were assembled in a custom-built Teflon cell and PDMS glued to prevent current leakage. Reservoirs on each side of the membrane were filled with a nanopore buffer containing 1 M KCl, 50 mM phosphate buffer, and 5 mM EDTA at different pHs, as indicated. All buffers were filtered using a 0.02-µm syringe filter and degassed in vacuum right before use, and the pH of the buffer was verified immediately before the experiment. All experiments were performed inside a dark Faraday cage at constant temperature (22.0 ± 0.5 °C). Two Ag wires were electroplated to form AgCl electrodes for both chambers, which were connected to an Axon 200B amplifier (Molecular Devices) that applied a voltage clamp as specified across the membrane. The ionic current flowing through the pore was measured, low pass filtered at 100 KHz and analyzed using a custom LabView (National Instruments) code, which saved each and every current blockade event over time. The translocation events were analyzed and the ion current amplitude ∆G (the difference in the conductivities between the blocked and open pore) and the pore dwell-time (t_D_) were determined.

### Nanopore verification

In order to verify that a drilled chip can be used for nanopore measurements, an I-V curve was generated at the beginning of the experiment, in which the current was measured as a function of ramped voltage. Linearity between current and voltage in both negative and positive bias was verified. The pore’s thickness and diameter were verified using the open pore’s conductivity as well as the DNA translocation measurement, and confirmed with the electron micrograph of the pore obtained after drilling. For the full protocol and calculation, see SI.

### Reduction and digestion experiment

Reduction experiment was performed by addition of TCEP, which was diluted in water and neutralized by addition of concentrated KOH solution. For the plasmin digestion experiment, lyopolized plasmin powder is purchased from Sigma, reconstituted in water to the stock concentration of 0.1 µg µl^−1^ and then stored at −80 °C until use. Upon usage, the stock solution was introduced directly into the nanopore chamber to the final concentration of 10 nM.

### Data collection

The electrical current across the nanopore was collected using the National Instrument A/D data acquisition boards via custom LabView Software at 250 kHz and low-pass filtered at 100 kHz.

### Data analysis and statistics

To compare across multiple voltage bias, the event amplitude was reported as a change in conductivity from the open pore current, defined by the ratio of the current to the voltage. The absolute change in open pore current was used instead of fractional blocked level to be able to compare data across multiple pores which have varying diameters and thicknesses, hence different open pore currents. An event was defined by a drop of more than 1 nS below the open pore level, which lasted at least 16 µs before spontaneously returning to the open pore level. The time between the starting and ending points was defined as the translocation dwell time. The average event amplitude is the all-point average during the dwell time. The dwell time histograms were characterized by tail-fitting the data using exponential functions to reduce the effect of extremely fast events limited by the temporal band-width of our system. The event extraction and analysis were done in MATLAB and Igor Pro, respectively.

## Electronic supplementary material

Supporting Information
