# Supplementary material for: Sensing Native Protein Solution Structures Using a Solid-state Nanopore: Unraveling the States of VEGF
Source: Sci Rep. 2018 Jan 17;8:1017. doi: 10.1038/s41598-018-19332-y (PMC5772516; doi:10.1038/s41598-018-19332-y)
Supplement: Supplementary file 1 — Supporting Information [file 41598_2018_19332_MOESM1_ESM.pdf]

Supplementary Information

Sensing native protein solution structures using a solid-state nanopore: Unraveling the states of VEGF

Nitinun Varongchayakul[1], Diana Huttner[4], Mark W. Grinstaff[*1,2,3], and Amit Meller[*1,4]

Departments of [1]Biomedical Engineering, [2]Chemistry, and [3]Medicine, Boston University, Massachusetts, USA, 02215 [4]Faculty of Biomedical Engineering, Technion- Israel Institute of Technology, Haifa, Israel, 32000

CONTENTS **Page**

Evidences of VEGF dimer and monomer in bulk solution S3

Determination of a nanopore's geometry S5

Translocation of VEGF in comparison with DNA S6

Estimation of the linearized peptide's event amplitude S7

Estimation of the hydrodynamic diameter of the VEGF protein S8

Continuous pore current during the VEGF translocation S9

Voltage-dependent VEGF translocation dynamics S10

Example event traces of VEGF at pH 7.6 before and after TCEP S11

Example event traces of VEGF at pH 7.2 before and after TCEP S13

Crystal structure of proteins involved in the study S14

Determining the turnover rate of plasmin-digested VEGF reaction using nanopore S15

Continuous pore currents for plasmin experiment S17

The raw experimental result of VEGF concentration dependency S18

Additional data sets S19

References S22

Evidences of VEGF dimer and monomer in bulk solution:

Full-length gel electrophoresis image of VEGF before and after reduction with TCEP

Figure SI1 shows a denaturing gel (SDS-PAGE) of the recombinant VEGF used in this experiment. The stock solution of 20 µM VEGF was diluted to final concentration of 0.5 ug in 10 ul phosphate buffer saline solution at pH 7.5. In one sample, pH-neutralized TCEP was added into the solution to obtain the final concentration of 10 mM and allowed the disulfide reaction to occur for 15 min. Both solutions were then mixed with 5x sample buffer containing 10% SDS, 20% glycerol, and small trace of bromophenolblue and boiled for 5 min.

The gel electrophoresis was performed using 4-20% TGX precast gel (Biorad, USA) with Tris/Glycine/SDS running buffer in electrophoresis gel apparatus (Biorad, USA). The voltage was set at 200 V and run for 30 min. The gel was then stained using a silver-stain (Pierce, ThermoFisher Scientific, USA).

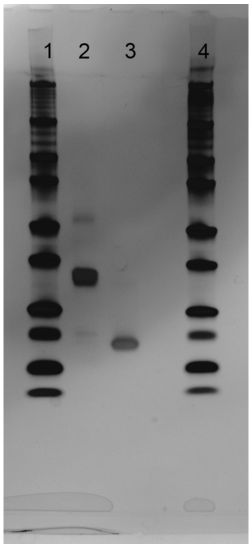

Figure SI 1. SDS-PAGE of VEGF, before and after interacting with TCEP. Silver stain. Lane1: 1 kb DNA Ladder (New England Biolabs, USA). Lane 2: 0.5 ug of VEGF without TCEP. Lane 3: 0.5 ug of VEGF with additional 10 mM of TCEP. Lane 4: 1 kb DNA Ladder.

Evidences of VEGF dimer and monomer in bulk solution: Mass spectroscopy result of VEGF

Matrix assisted laser desorption ionization-time of flight mass spectrometry (MALDI-TOF MS) was performed to detect the presence of monomer and dimer of VEGF at pH 6.8 and 8.0, as shown in figure SI1. Roughly, 1 ug of VEGF was diluted into 1 ul of 50 mM phosphate buffer at pH 6.8 and 8.0 and was equilibrated for 15 min (final concentration, 50 uM). The VEGF solution was then deposited onto a HCCA/1% TFA solution on a target plate and quickly dried using heating gun. The sample was then analyzed the mass spectra using MALDI-TOF MS technique (Bruker autoflex Speed, at 10-50 kDa window). The mass spectra consistently showed a mixed population of monomer, at 19-kDa peak and dimer at 38-kDa peak. We also observed that the ratio between two peaks were not the same in two different pH. Although we realized that the ionization process might artificially introduce more monomer, this result provided a supported evidence of a presence of monomer and dimer in a solution, and the ratio was varied as a function of pH's solution.

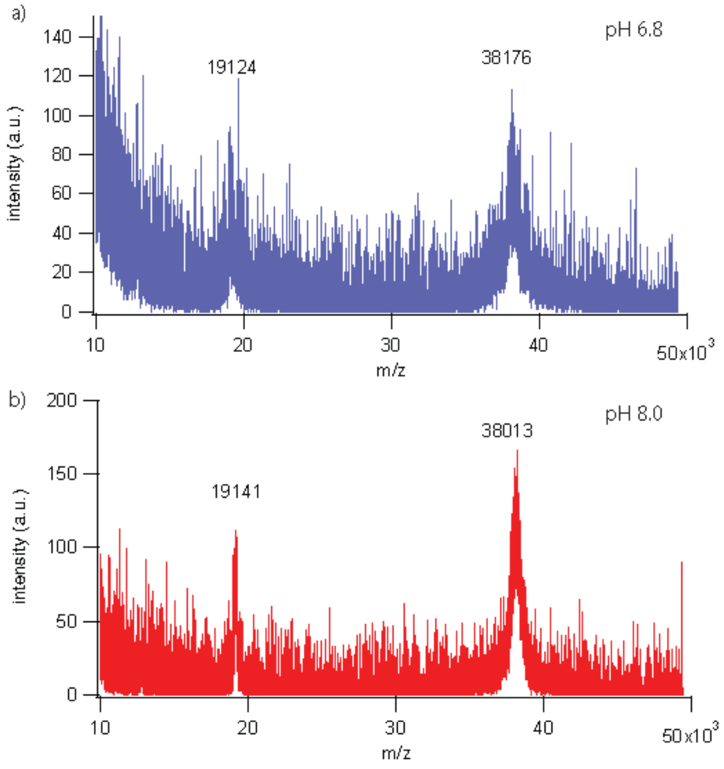

Figure SI 2. Representative MALDI-TOF-MS spectra of 5uM VEGF pre-equilibrated in PBS at (a) pH 6.8 and (b) pH 8.0 to confirm the molecular weight of the two proteins.

Determination of a nanopore's geometry

We evaluate the pore's geometry (thickness and diameter) by first using the measured parameters obtained from the fabrication process and later on confirmed with the conductance measurement. Initially, the film thickness was determined by the elipsometer. With a known etching rate, the locally thinned area thickness was calculated. After the nanopore was formed by mean of the transmission electron microscope (TEM), the electron micrograph of the pore was taken and used to measure the diameter of the pore. We anticipate that after the pore went through intensive cleaning with piranha solution, the final geometry might changes, thus the conductance measurement was performed before the protein translocation experiment. The dsDNA was translocated through a nanopore and the open pore current as well as the blocked current were measured. The two set of equations, which derived from a geometrical model of pore conductance, were used to fit the two unknowns (thickness and diameter)[1].

$$G_o = \sigma \left[\frac{4l}{\pi (d_p)^2} + \frac{1}{(d_p)}\right]^{-1} \qquad \text{(eq.S1)}$$

And

$$G_o - \Delta G = \sigma \left[\frac{4l}{\pi (d_{eff})^2} + \frac{1}{(d_{eff})}\right]^{-1},\ d_{eff} = \sqrt{d_p^2 - d_M^2} \qquad \text{(eq.S2)}$$

Where, ΔG and G_o are the event amplitude and open pore current respectively. l is length of the pore, d_{eff} is the effective diameter of the pore when the pore diameter $\mathrm{d_p}$ is partly occupied by the molecule diameter d_M. σ is the buffer solution conductivitiy (10.5 nS nm^{-1} for 1M KCl, 25°C). Using this model, the pore we used in this study was found to have a thickness, l = 12 nm and d_p =6.0 nm.

Translocation of VEGF in comparison with DNA

As a control, and to ensure that the nanopore itself did not bias the translocation pattern of the VEGF, DNA translocation experiment was performed to verify the pore's geometry and its performance. After VEGF translocation experiment, we washed the *cis* chamber with clear buffer and immediately measured the translocation of 5 kbps dsDNA. As expected, the dsDNA produced a single cluster of events (Figure 1b, right panel) with event amplitude of -1.5±0.3 nS (N = 616) and a characteristic dwell time of 75±15 μs, in agreement with the TEM-measured pore diameter and in accord with previous findings [2].

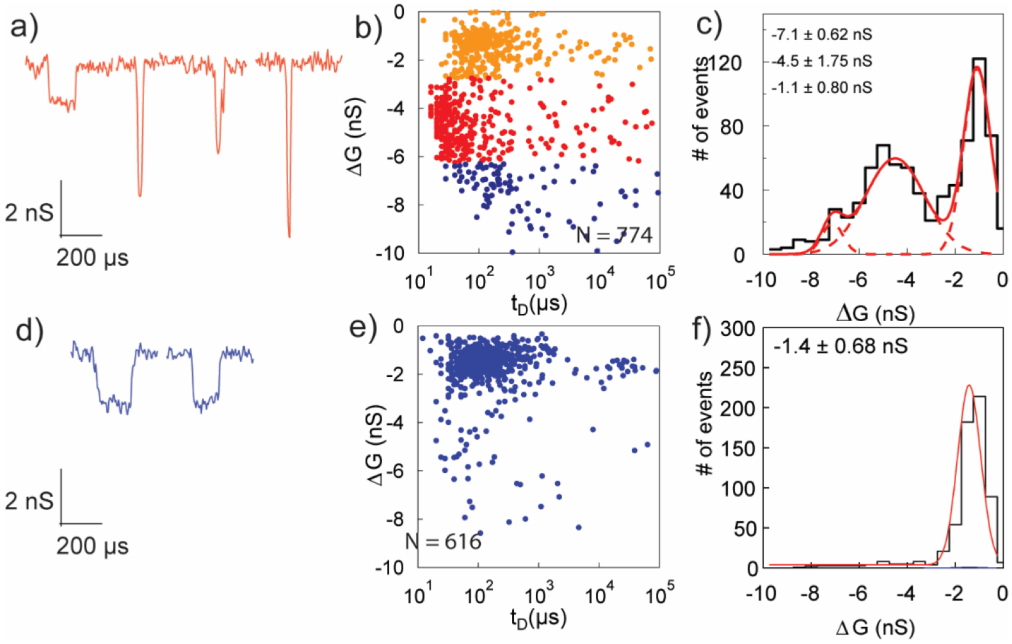

Figure SI 3. (a) The translocation behavior of VEGF. (b) scatter plot of VEGF translocation shows three distinctive populations (different color). (c) Amplitude histogram of VEGF translocation events. (d-f) The translocation of 5 kbps DNA was also performed for comparison. The experiment was done in the same 6.0 nm diameter pore and 12 nm thickness at pH 7.2 and voltage bias of +300 mV.

Estimation of the linearized peptide's event amplitude

If VEGF subjected to be unfolded and linearized, it would have a length of 85 nm (estimated by bond length of 165 amino acids) and approximate cross section 0.45 nm [3]. Since unfolded peptide translocated as a linear chain, the relationship between the event amplitudes and cross section of the molecule is followed[4]:

$$\frac{\Delta G_{peptide}}{\Delta G_{DNA}} = \left(\frac{d_{peptide}}{d_{DNA}}\right)^2 \qquad \text{(eq.S3)}$$

This equation give an expected block current of a linearized peptide of 0.2 nS, which is smaller than the value obtained in the experiment by a factor of 10. Therefore, it is unlikely that VEGF is translocated through the nanopore as a fully linearized peptide under our experimental condition.

Estimation of the hydrodynamic diameter of the VEGF protein.

To determine the hydrodynamic diameter of the VEGF protein, d_H, we first estimate the theoretical smallest size of the protein assuming that it is a closed-pack spherical particle according to Erickson's formula[5]

$$d\_\min = 2r_{min} = 0.132M^{\frac{1}{3}} \quad \text{(eq.S4)}$$

for M in Dalton and d_{min} in nanometer. Then assuming the shape correction factor of 1.2 as VEGF is relatively spherical shape and experimentally determined by sedimentation analysis[6], the hydrodynamic radius is 4.12 nm and 5.20 nm for VEGF monomer and dimer respectively.

This value is in the same scale as when the hydrodynamic radius is calculated from crystal structure of VEGF (pdb file: 1vpf) using an algorithm named HYDROPRO[7], which yields R_H = 6.32 nm for VEGF dimer.

Continuous pore current during the VEGF translocation

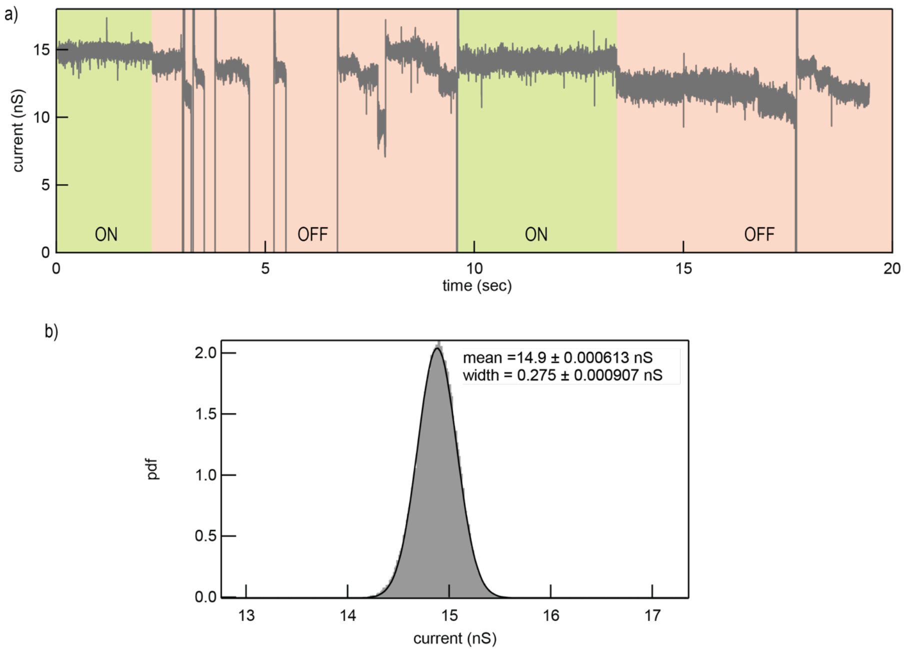

Figure SI 4. (a) The pore current during VEGF translocation at pH 7.6. While the apparent event capture rate is high (~0.03 molecule sec^{-1} nM^{-1}), the pore subjected to long-timescale reduction in open pore current which we contribute to the non-specific adsorption of a protein onto the pore's surface. For consistency, we discarded the data where the open pore current fluctuated (denoted as OFF in the graph). A typical time for data collection in each experimental is 10 – 15 min. (b) The histogram of the open pore current fitted with Gaussian curve.

Voltage-dependent VEGF translocation

In order to verify that most of the ion current blockade events correspond to protein translocation and not collisions, we measured the dwell-time histogram of VEGF under different voltages ***using the same nanopore***. Briefly, VEGF was introduced into a nanopore chamber containing 1 M KCl, 50 mM phosphate buffer at pH 7.2 and 5 mM EDTA such that the final concentration of the protein is 20 nM. The voltage bias was set at -300 mV on the *trans* (opposite) chamber to attract positively charged VEGF. After 10 minutes of data collection, the voltage bias was changed to -500 mV, data was collected for another 10 minutes and same procedure was repeated at -700 mV. Figure SI5 shows the result of this voltage-dependent experiment.

As the absolute value of voltage increases from 300 to 700 mV, the mean residence time (obtained from fitting the dwell time histogram with the exponential functions) *decreases* from 391±55 μs to 56±4 μs. Notably, if the observed events were due to protein collisions, increasing voltage should result in longer dwell time[4]. This experiment thus negates this possibility, supporting the hypothesis that the observed events likely result from the translocation of VEGF.

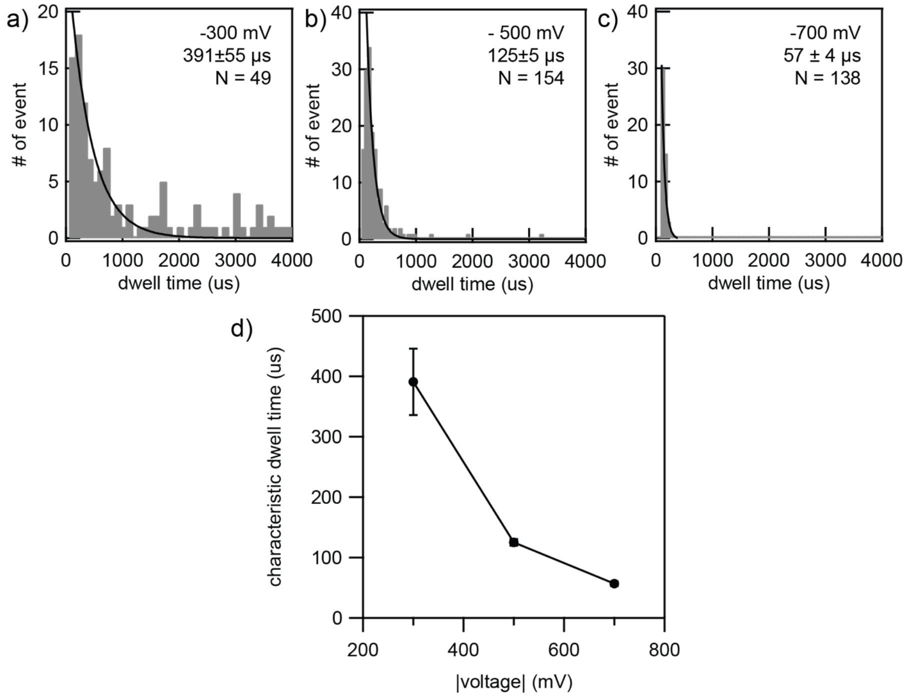

Figure SI 5. Dwell time histograms of the VEGF translocation at different voltage bias show a sharp decrease in the characteristic dwell time as a function of voltage. (a- c) The histograms are tail-fitted with the exponential functions (~exp(-t/T_D) function to determine the characteristic dwell time. (d) The characteristic dwell time as a function of applied voltage. The three data set were collected fusing the same nanopore (12 nm thickness and 5.5 nm diameter).

Example event traces of VEGF at pH 7.6 before and after TCEP

Here we showed additional translocation events from VEGF at pH 7.6 before introducing TCEP.

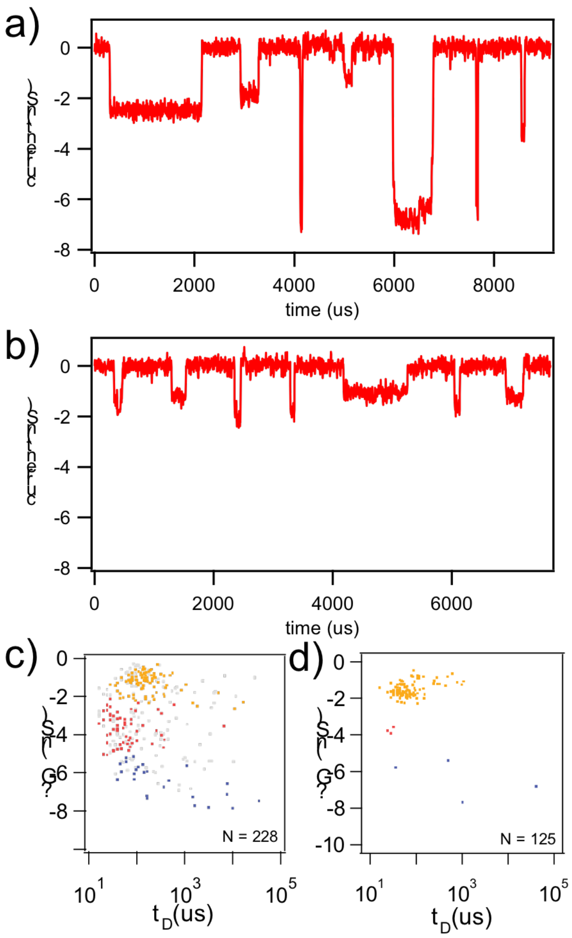

Figure SI 6. (a) Example event traces of 20 nM VEGF at pH 7.6, (b) after introducing 0.5 mM of TCEP. (c) The scatter plot of VEGF translocation at pH 7.6. There are 3 distinctive group of events classified by their amplitude. Orange, red, and blue represent group A, B and C respectively. The grey dots represent segmented levels from multi-level events. For instant, a multi-level with 2 sublevels contributes to 2 grey dots. (d) The scatter plot of VEGF translocation after introducing TCEP.

Example event traces of VEGF at pH 7.2 before and after TCEP

Here we showed additional translocation events from VEGF at pH 7.2 (figure SI4a). One can see that the event that exhibits multiple steps mostly in B to A transition pattern. Different level is clearly distinguished and larger than the noise floor. After introducing TCEP (figure SI4b), the majority of the events only show single level pattern.

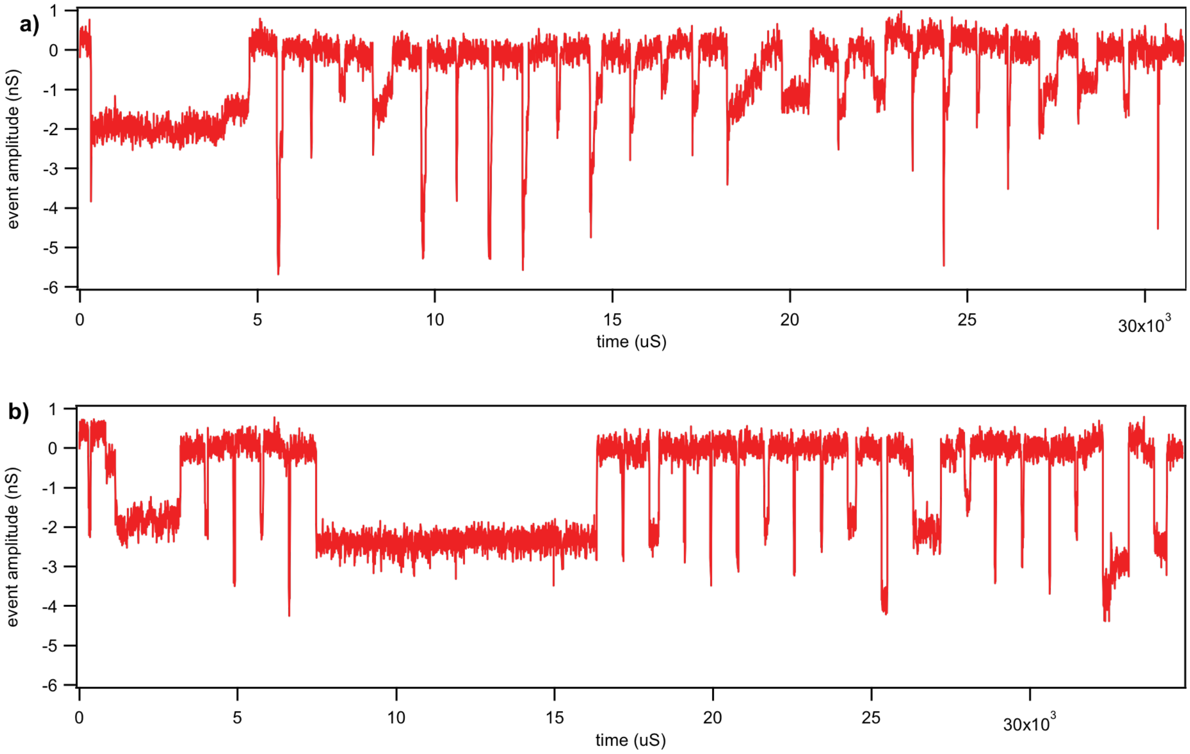

Figure SI 7. Example event traces of 20 nM VEGF (a) at pH 7.2, (b) after introducing TCEP.

Crystal structure of proteins involved in the study

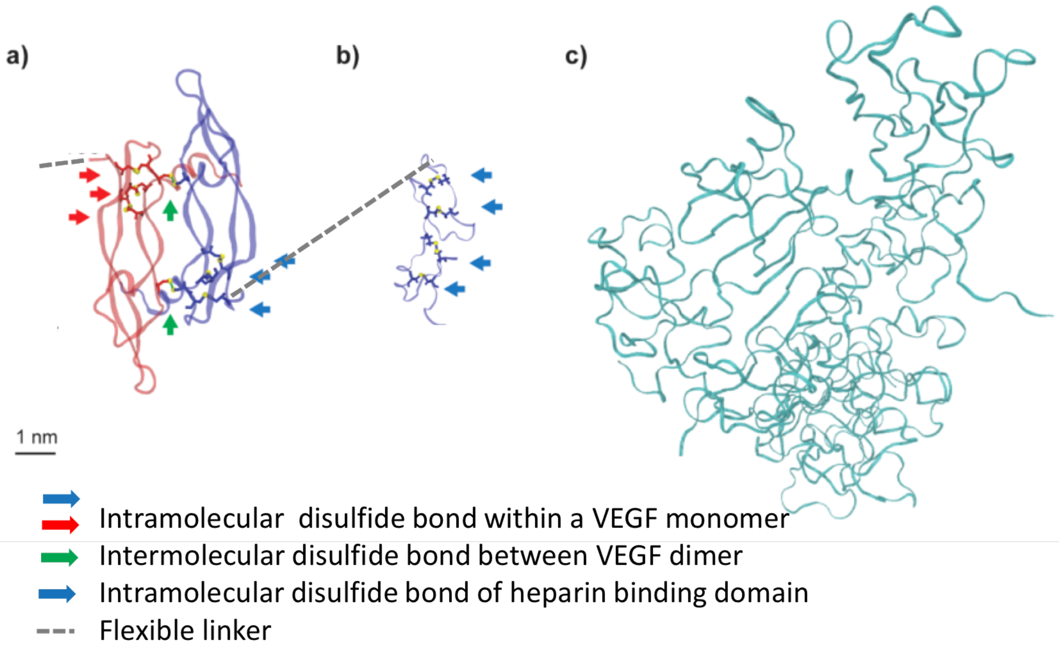

Figure SI 8. Crystal structures of the protein used in this experiment. (a) VEGF receptor recognition (body) domain. A monomer is shown in red and another monomer is shown in blue. Three disulfide bonds which stabilize the internal structure by forming cysteine knots are indicated by red and blue arrows for each monomer. Two disulfide bonds that involved in dimerization are indicated by green arrows. The residue numbers are labelled. The crystal structure is obtained from pdb file 2vpf and rendered in VMD. (b) Heparin binding (tail) domain consists of four disulfide bonds that stabilize the internal structures, indicated by blue arrows. The crystal structure is obtained from pdb file 1vph. (c) Human plasmin (ogen) used for cleaving VEGF body and tail domains for size comparison. The crystal structure is obtained from pdb file 4ddu and rendered in VMD.

Determining the turnover rate of plasmin-digested VEGF reaction using nanopore

From figure 5 in the main manuscript, we determined the VEGF turnover rate with plasmin to have a half-life of ~10 min. To obtain the reaction rate constant, we first assume that the rate-limiting step of the reaction is the first process, where $VEGF_{165}$ is cleaved into $VEGF_{1\text{-}110}$ and $VEGF_{111\text{-}165}$;

$$VEGF_{165} + Plasmin \rightarrow VEGF_{1\text{-}110} + VEGF_{111\text{-}165} + Plasmin$$

This sequence follows the first order kinetics as shown by Vempati et.al. [8],

$$\frac{d[VEGF_{165}]}{dt} = -k_p[plasmin][VEGF_{165}]$$

From this model, the half-life is related to the reaction constant, k_p, and plasmin concentration by

$$t_{\frac{1}{2}} = \frac{0.693}{k_p[plasmin]}$$

Under our experimental condition (10 nM plasmin), the reaction constant is $1.6 \cdot 10^5\ M^{-1}s^{-1}$. This kinetic parameter is representative of high end of extracellular matrix protease reactions and two order of magnitude higher than the experimental value in bulk $5 \cdot 10^3\ M^{-1}s^{-1}$ (see table below). We expect that our rate constant will be higher since our sample lags of glycosylation, hence the cleavage site is more exposed to plasmin digest.

Substrate	Protease	Kp	temperature	Ref.
collagen	MMP-1	$900 \cdot 10^3\ M^{-1}s^{-1}$	30°C	[9]
Type I collagen	collagenase	$120 \cdot 10^3\ M^{-1}s^{-1}$	37°C	[10]
Glycosylated VEGF	Plasmin	$0.6 \cdot 10^3\ M^{-1}s^{-1}$	25°C	[11]
Glycosylated VEGF	plasmin	$5 \cdot 10^3\ M^{-1}s^{-1}$	37°C	[12]

Table SI 1. The reported reaction rate constants of various protease systems

Continuous pore currents for plasmin experiment

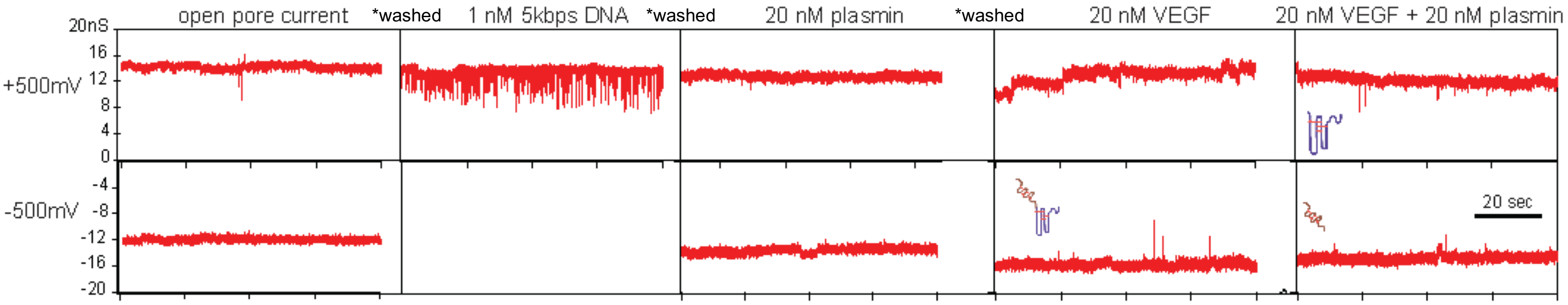

Figure SI 9. Representative continuous current traces of the pore during the plasmin cleavage experiment. The continuous current was collected at 25 kHz and filtered using moving average (bin of 3) for visualization. After each experiment was done, the pore was thoroughly washed with nanopore buffer 3 times and make sure that there was no event observed before introducing a new analyst. All data is collected in the same pore consecutively (diameter 4.5 nm, thickness 7 nm, pH 7.2)

The raw experimental result of VEGF concentration dependency

no	conc (nM)	pH	diameter (nm)	thickness (nm)	voltage (mV)	total number of events	A	B	C	A/B	else	%A	%B + B/A
1	0.2	7.2	4	7	500	54	38	16	0	0	0	70	30
2	10	7.2	6	20	500	153	115	10	16	5	7	75	10
3	20	7.2	4.5	12	500	73	33	20	2	11	7	45	42
4	20	7.2	4	12	500	80	39	12	0	22	7	49	43
5	20	7.2	4	12	500	104	43	24	9	5	23	41	28
6	40	7.2	6	12	500	130	50	42	7	22	9	38	49
7	40	7.2	4	12	500	130	54	35	6	22	13	42	44
8	50	7.2	6.5	12	500	103	14	48	21	6	14	14	52
9	100	7.2	6.5	12	500	212	21	88	7	37	59	10	59
10	100	7.2	4.2	7	400	246	16	85	0	118	27	7	83
11	100	7.2	4.2	7	400	168	17	62	0	61	28	10	73
12	5	7.2	6.3	12	300	42	37	0	0	5	0	88	12
13	10	7.2	6	12	300	47	40	1	2	0	4	85	2

Table SI 2. The experimental result of VEGF translocation at different concentration which is used to generate the figure 4 in the main manuscript

Additional data sets

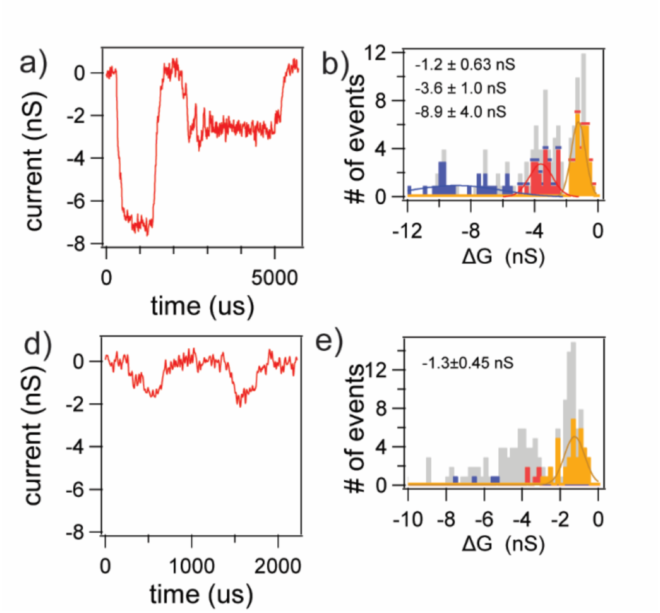

Figure SI 10. Additional data set for VEGF translocation experiment at pH 7.6 (a,b) and with addition of TCEP (d,e). The experiment was done in 6.5 nm diameter, 12 nm thickness pore at +500 mV.

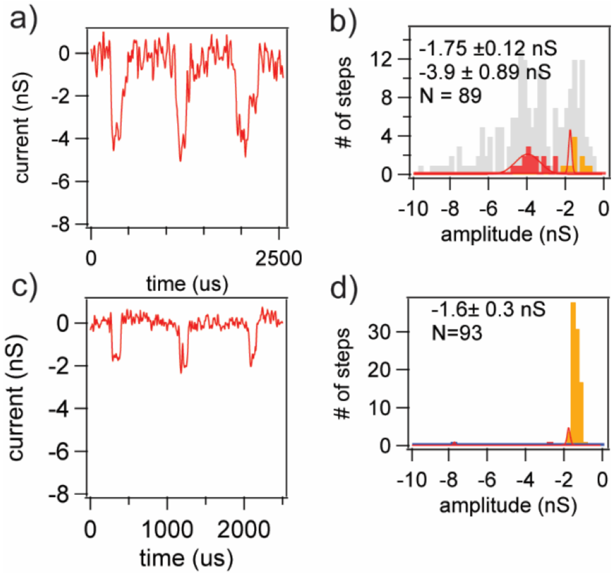

Figure SI 11. Additional data set for VEGF translocation experiment at pH 7.2 (a,b) and with the addition of TCEP (c,d). The experiment was done in 6.5 nm diameter, 12 nm thickness pore at -300 mV.

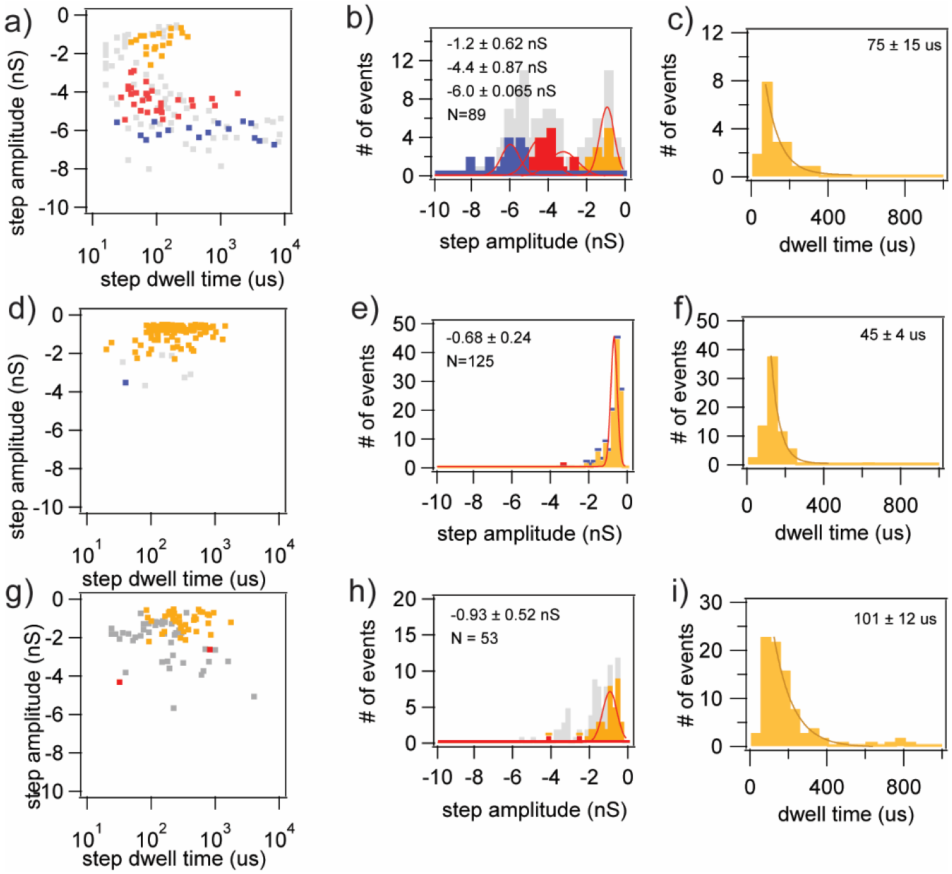

Figure SI 12. An additional experiment for plasmin cleavage reaction. (a) The scatter plot of the amplitude vs dwell time of the VEGF translocation events at pH 7.2 probed at -500 mV. (b) The event amplitude histogram and (c) dwell time histogram of VEGF translocation events. (d-f) The scatter plot, amplitude histogram and dwell time histogram of VEGF, 15 min after addition of plasmin. The translocation events were probed under the negative bias. (g-i) The translocation events after addition of plasmin, probed under the positive bias (+500 mV). The entire set of the experiment were done in the same 5.0 nm pore.

References

1. Hyun, C., Rollings, R. & Li, J. Probing Access Resistance of Solid-State Nanopores with a Scanning-Probe Microscope Tip. *Small* **8,** 385–392 (2012).

2. Wanunu, M. *et al.* Rapid electronic detection of probe-specific microRNAs using thin nanopore sensors. *Nat. Nanotechnol.* **5,** 807–814 (2010).

3. Talaga, D. S. & Li, J. Single-molecule protein unfolding in solid state nanopores. *J. Am. Chem. Soc.* **131,** 9287–97 (2009).

4. Wanunu, M., Sutin, J., McNally, B., Chow, A. & Meller, A. DNA translocation governed by interactions with solid-state nanopores. *Biophys. J.* **95,** 4716–25 (2008).

5. Erickson, H. P. Size and shape of protein molecules at the nanometer level determined by sedimentation, gel filtration, and electron microscopy. *Biol. Proced. Online* **11,** 32–51 (2009).

6. Yang, J. *et al.* Comparison of Binding Characteristics and In Vitro Activities of Three Inhibitors of Vascular Endothelial Growth Factor A. *Mol. Pharm.* **11,** 3421–3430 (2014).

7. Ortega, A., Amorós, D. & García de la Torre, J. Prediction of hydrodynamic and other solution properties of rigid proteins from atomic- and residue-level models. *Biophys. J.* **101,** 892–8 (2011).

8. Vempati, P., Mac Gabhann, F. & Popel, A. S. Quantifying the Proteolytic Release of Extracellular Matrix-Sequestered VEGF with a Computational Model. *PLoS One* **5,** e11860 (2010).

9. Lauer-Fields, J. L. *et al.* Kinetic analysis of matrix metalloproteinase activity using fluorogenic triple-helical substrates. *Biochemistry* **40,** 5795–803 (2001).

10. Seltzer, J. L. *et al.* Cleavage specificity of type IV collagenase (gelatinase) from human skin. Use of synthetic peptides as model substrates. *J. Biol. Chem.* **264,** 19583–6 (1989).

11. Keck, R. G., Berleau, L., Harris, R. & Keyt, B. A. Disulfide structure of the heparin binding domain in vascular endothelial growth factor: characterization of posttranslational modifications in VEGF. *Arch. Biochem. Biophys.* **344,** 103–13 (1997).

12. Lauer, G., Sollberg, S., Cole, M., Krieg, T. & Eming, S. A. Generation of a novel proteolysis resistant vascular endothelial growth factor165 variant by a site-directed mutation at the plasmin sensitive cleavage site. *FEBS Lett.* **531,** 309–13 (2002).
